# Detectability of subsegmental lesions in patients with inoperable CTEPH: Comparison between ultra-high-resolution vs. conventional CT

**DOI:** 10.1016/j.jhlto.2025.100344

**Published:** 2025-07-18

**Authors:** Satoshi Higuchi, Taijyu Satoh, Hidenobu Takagi, Mitsuru Nakada, Takuya Kawahara, Nobuhiro Yaoita, Shuhei Sugiyama, Tomoya Onuma, Kenta Shirata, Shingo Kayano, Hideki Ota, Satoshi Yasuda, Kei Takase

**Affiliations:** a Department of Diagnostic Radiology, Tohoku University Hospital, Sendai, Japan; b Department of Cardiovascular Medicine, Tohoku University Graduate School of Medicine, Sendai, Japan; c Department of Radiology, Tohoku University Hospital, Sendai, Japan; d Clinical Research Promotion Center, The University of Tokyo Hospital, Bunkyo, Japan; e Department of Pulmonary Medicine, Amsterdam UMC, Amsterdam, The Netherlands

**Keywords:** CTEPH, BPA, Ultra-high-resolution CT, Subsegmental lesion

## Abstract

**Background:**

CT pulmonary angiography (CTPA) plays a critical role in guiding balloon pulmonary angioplasty (BPA) for patients with chronic thromboembolic pulmonary hypertension (CTEPH). However, conventional CT (cCT) has limited sensitivity in detecting peripheral lesions, which is critical for avoiding complications. This study compared ultra-high-resolution CT (UHRCT; 0.25 mm detector elements) and conventional CT (cCT; 0.6 mm detector elements) in identifying and classifying segmental and subsegmental lesions, using invasive selective angiography during BPA as the reference standard.

**Methods:**

This single-center retrospective study included 42 patients with newly diagnosed CTEPH who underwent CT pulmonary angiography (CTPA) with either cCT or UHRCT and subsequently completed BPA. The morphology and location of lesions were independently assessed using selective angiography and CTPA. Sensitivity, specificity, and lesion classification accuracy were assessed using selective angiography as the reference standard.

**Results:**

A total of 1687 branches in 42 patients (male/female 11/31, mean age 66 years) were analyzed. The sensitivity and specificity of cCT were 54.6% (95% CI: 48.2–60.8) and 85.2% (95% CI: 75.6–91.4), respectively. In contrast, UHRCT demonstrated significantly higher sensitivity (94.3%, 95% CI: 91.9–96.1) but lower specificity (60.2%, 95% CI: 46.7–72.2). The sensitivity difference was more prominent in subsegmental branches (p for interaction = 0.11). UHRCT more accurately classified lesion types in 83.7% of cases (95% CI: 76.7–88.9), versus 69.1% (95% CI: 58.3–78.1) with cCT. Web lesions remained the most difficult to detect.

**Conclusion:**

Higher-spatial-resolution CTPA provides a higher lesion detection sensitivity, particularly in subsegmental branches, and more accurately classified lesion type in patients with CTEPH treated with BPA, potentially aiding procedural planning and guidance.

## Introduction

Chronic thromboembolic pulmonary hypertension (CTEPH) arises from unresolved thrombi that organize into fibrous tissue, obstructing the pulmonary arteries and leading to right heart failure if left untreated.^1^, ^2^ Balloon pulmonary angioplasty (BPA) is an interventional treatment to reopen stenotic or occluded vessels in segmental or subsegmental branches that are inaccessible via pulmonary endarterectomy (PEA).^1^, ^2^ Although multimodal treatments have significantly improved the prognosis for CTEPH patients,^3^, ^4^, ^5^ bleeding complications during BPA can still pose life-threatening risks.^6^ The success and complication rates of BPA are closely tied to the lesion's morphologic characteristics, highlighting the importance of establishing preoperative strategies to optimize outcomes and minimize risks.^7^

CT imaging plays a crucial role in offering rapid scanning, high spatial and temporal resolution, and comprehensive evaluation of cardiopulmonary structures in managing pulmonary hypertension.^8^ High-volume centers often use CT imaging to map the extent and location of lesions, aiding in the assessment of operability and guiding working projections during BPA.^9^, ^10^ However, invasive pulmonary angiography remains the gold standard for evaluating peripheral vessel lesions due to the limited spatial resolution of conventional CT pulmonary angiography (CTPA).^9^, ^11^ Ultra-high-resolution CT (UHRCT), with smaller detector elements and x-ray tube focus size, surpasses the spatial resolution of conventional CT, allowing for enhanced visualization of fine arterial structures and lesions.^12^ We hypothesized that UHRCT could improve the detection of peripheral pulmonary artery lesions in CTEPH patients undergoing BPA, ultimately enhancing preoperative planning and outcomes. The purpose of this study was to compare the detectability of lesions in segmental and subsegmental pulmonary arteries using UHRCT versus conventional CT, as validated by invasive selective angiography during BPA in patients with CTEPH.

## Materials and methods

### Study design and participants

This is a single-center observational study conducted retrospectively in accordance with the Declaration of Helsinki (Washington, World Medical Association, 2013) and approved by the research ethics committee (approval #2023–1-050). Given the retrospective design, written informed consent was not obtained from patients. The study included 54 consecutive patients with newly diagnosed CTEPH between 2018 and 2021. Diagnosis of CTEPH and treatment planning were conducted through multidisciplinary team discussions involving cardiologists, radiologists, and interventional radiologists. Patients who were not treated with BPA, had iodine contrast allergy, did not complete BPA, or underwent CTPA at other hospitals were excluded. In this study we will report patient characteristics, number of BPA sessions, hemodynamic parameters before BPA.

### CTPA acquisition and reconstruction protocol

CTPA performed at the time of diagnosis was analyzed to identify lesions in the pulmonary arteries, blinded to the angiographic findings during BPA. Until August 2019, CTPA was performed with 2nd-generation dual-source CT (SOMATOM Definition Flash, Siemens Healthineers, Forchleim, Germany) and dual-energy CT (DECT) was acquired after a test injection scan with parameters in Table 1. The images were reconstructed using a hybrid iterative reconstruction algorithm (Sinogram Affirmed Iterative Reconstruction: SAFIRE), with following parameters: slice thickness = 1 mm, matrix size = 512, field of view (FOV) = 330 (305−345) mm, and estimated pixel size = 0.64 (0.59–0.67) mm. Since the installation of UHRCT (Aquilion Precision; Canon medical systems, Otawara, Japan) in August 2019, CTPA was performed using UHRCT with the parameters in Table 1. Images were reconstructed using a deep learning reconstruction algorithm (Advanced intelligent Clear-IQ Engine: AiCE) with following parameters: slice thickness = 0.25 mm, matrix size = 1024, FOV = 320 (300−350) and estimated pixel size = 0.31 (0.29–0.34) mm. These images were evaluated using a designated workstation (Ziostation2; Ziosoft, Tokyo, Japan).

**Table 1:** Scan and Reconstruction Parameters

	Conventional CT	Ultra-high-resolution CT
Scan		
CT scanner	SOMATOM Definition Flash	Aquilion Precision
Tube voltage (kV)	80 / Sn140	120
Reference tube current (mAs)	141	NA
Noise index (standard deviation)	NA	10 @ 5 mm
Collimation	0.6 mm * 64	0.25 mm * 160
Beam pitch factor	0.55	1.381
Rotation speed (sec)	0.28	0.35
Direction of image acquisition	caudocranial	caudocranial
Scan timing	end-inspiratory	end-inspiratory
Injection		
Injector	Dual Shot GX7	Dual Shot GX7
Scan timing	Test injection	Fixed (17 s)
Concentration of iodine	350 (350−350)	350 (350−350)
Injection rate (mgI/kg/sec)	26	26
Injection duration (sec)	6	16
Reconstruction		
Dual energy composition	0.6	NA
Kernel	SAFIRE (I30f)	AiCE (body sharp standard)
Thickness (mm)	1	0.25
Slice increment (mm)	1	0.25
Matrix	512	1024
Display field of view size (mm)	330 (305−345)	320 (300−350)
Estimated pixel size (mm)	0.64 (0.59−0.67)	0.31 (0.29−0.34)

AiCE, advanced intelligent clear-IQ engine; SAFIRE, sinogram affirmed iterative reconstruction

### Selective angiography during BPA

BPA was performed by a collaborative team of interventional cardiologists and radiologists. To ensure a consistent treatment strategy, multidisciplinary discussions were held prior to each procedure. BPA was performed under angiographic guidance in staged sessions, typically starting with the lower lobes, followed by the middle, and then upper lobes. BPA was continued until the mean pulmonary arterial pressure (mPAP) decreased to below 25 mmHg or pulmonary vascular resistance (PVR) decreased to below 300 dynes·sec·cm⁻⁵. In BPA, target vessels were selected using a 6-Fr guiding catheter with a 6-Fr long sheath. The lesions were evaluated by the segmental angiography with a power injector from two point of views using dual plane fluoroscopy system (INFX-8000V/JT, Canon Medical Systems, Otawara, Japan). The angle was determined at beam incidence angle as perpendicular as possible to running of the target vessel.

### Image analysis

**Figure 1:**
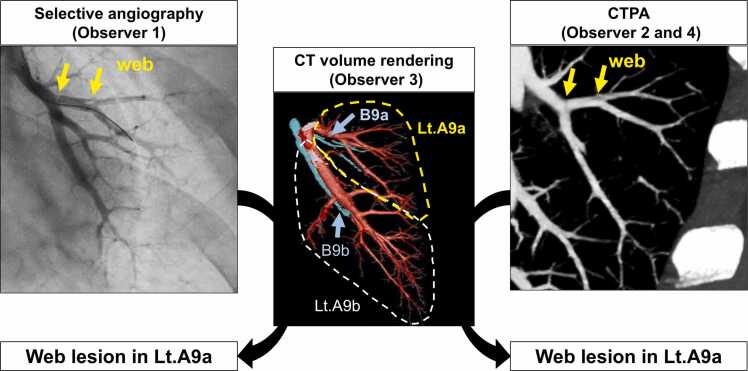
Evaluation of lesions in each modality. Web lesions were marked on selective angiography by a cardiologist (observer 1) and on CT pulmonary angiography (CTPA) by a radiologist (observer 2), independently (yellow arrows). Segmental and subsegmental branches were defined based on the anatomical features of the bronchus on the CT volume rendering images. Observer 3 identified the branches with these lesions on selective angiography and CTPA images using this classification, indicating web lesions were detected in left A9a on each modality. Observer 4 also evaluated CTPA images in 12 randomly selected patients from each group to assess interobserver reproducibility.

The morphology and location of lesions were independently marked by a cardiologist (observer 1; T.S., 13 years of experience, including 8 years of expertise) on selective angiography, and by a radiologist (observer 2; S.H., 10 years of experience, including 5 years of expertise) on axial CTPA images, blinded to lung perfusion maps. CT images were reviewed in chronological order, as blinding to scanner type was not feasible due to the distinguishable image quality between cCT and UHRCT. To minimize potential bias, both reviewers predefined borderline or subtle lesions as ‘no lesion,’ as these cases were also difficult to classify morphologically. Lesions were classified into four morphological types: ringlike stenosis, web, subtotal occlusion, and total occlusion.^13^ Ringlike stenoses and webs were further classified as obstructive lesions, while subtotal occlusions and total occlusions were classified as occlusive lesions. Segmental and subsegmental branches were defined by the anatomical feature of the bronchus,^14^ and each branch was classified using CT volume rendering images. Based on this classification, a third observer (M.N. with 20 years of experience and 12 years of expertise) identified the branches with lesions on selective angiography and CTPA images (Figure 1). To assess interobserver reproducibility, a radiologist (H.T., 13 years of experience and 8 years of expertise) and a radiological technologist (M.N., 12 years of expertise in BPA) independently evaluated CTPA images and selective angiography in all patients. To evaluate intraobserver reproducibility, one reviewer reassessed CT images from 10 randomly selected patients in each group after an interval of more than one year, blinded to the initial evaluations. Finally, CT dose index (CTDI), dose length product (DLP), and CT values and its standard deviation (SD) in pulmonary arterial trunk, ascending aorta, and air outside the body were evaluated.

### Statistical analyses

Descriptive statistics are presented as means and standard deviations (s.d.) for continuous variables and as the number of cases and percentages per group for categorical variables. Nonparametric data are shown as medians with interquartile ranges (IQRs). The normality of parameters and patient demographic data were tested using the Shapiro-Wilk test. Variables between the two groups were compared using Student’s t-test or Wilcoxon/Kruskal-Wallis test. For dichotomous data, Fisher’s exact test was used to analyze the difference in proportions between the groups. We assessed the sensitivity and specificity of each CT scanner, and the agreement rates of lesion classification between CTPA and angiography using a generalized linear mixed models (GLMM) with a logit link function to account for patient-level correlations. We employed logistic regression with random effects with scanner type as a fixed effect and patient identification number as a random interception. The presence of interactions between scanner type and lesion location was also tested. Additionally, Free-Response Receiver Operating Characteristic (FROC) analysis was performed to evaluate lesion detection performance. Lesion detection sensitivity differences between cCT and UHRCT at a predefined non-lesion detection (false positive) threshold (0.1) were evaluated. The 95% confidence interval (CI) of the lesion detection sensitivity difference was estimated using the bootstrap percentile method based on 1000 resampling at patient-level. We also assessed the morphological types of lesions undetected by CTPA. Cohen’s kappa (κ) statistics were used to assess interobserver agreement between two independent readers for both CT-based and angiography-based lesion detection, as well as intraobserver agreement between two evaluations by the same CT reviewer at different time points. To address the limitation of Cohen’s Kappa statistics, which tends to be attenuated to zero when the number of cases with or without lesions are highly imbalanced, we also calculated Cohen’s Kappa for a balanced dataset by randomly sampling from the majority class to match the number of observations in the minority class. The Kappa was interpreted as follows: values ≤ 0 as indicating no agreement and 0.01–0.20 as none to slight, 0.21–0.40 as fair, 0.41– 0.60 as moderate, 0.61–0.80 as substantial, and 0.81–1.00 as almost perfect agreement.^15^ Statistical analyses were performed using R version 4.3.2. P-values < 0.05 were considered statistically significant. In the analysis of the effect of interaction, p-values < 0.2 were considered statistically significant.^16^

## Results

### The patients background and evaluated vessels

**Figure 2:**
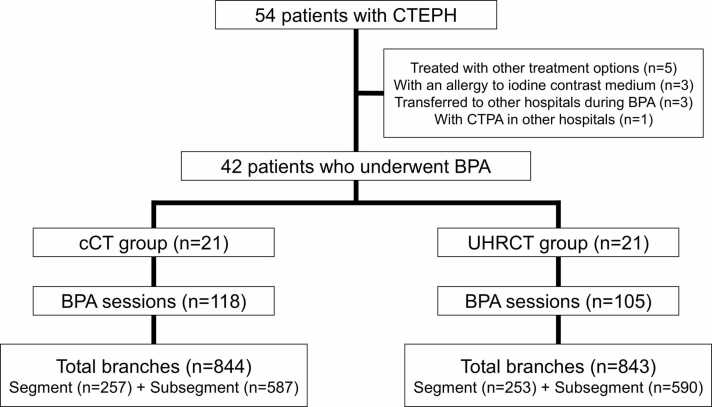
Flow diagram of study participants**.** The fifty-four consecutive patients with newly diagnosed chronic thromboembolic pulmonary hypertension (CTEPH) from 2018 to 2021 were included in this study. The patients treated with other treatment options (n=5), patients with allergies for iodine contrast medium (n=3), patients transferred to other hospitals during balloon pulmonary angioplasty (BPA) (n=3), a patient who underwent CTPA in other hospital were excluded. In 42 patients, 21 patients underwent CTPA with conventional CT (cCT) and other half underwent CTPA with ultra-high-resolution CT (UHRCT). The cCT and UHRCT group experienced 118 and 105 BPA sessions, respectively. A total of 844 branches (256 segmental and 587 subsegmental) were evaluated in the cCT group, while 843 branches (253 segmental and 590 subsegmental) were evaluated in the UHRCT group.

Out of 54 patients, 12 were excluded from the analysis, resulting in a total of 42 patients being included (Figure 2). Twenty-one patients underwent CTPA with cCT, and 21 with UHRCT. A total of 844 branches (256 segmental and 587 subsegmental) were evaluated in the cCT group, while 843 branches (253 segmental and 590 subsegmental) were evaluated in the UHRCT group (Figure 2). The distribution of evaluated branches is detailed in the Supplemental Figure, showing no significant difference in distribution between the two groups (p = 0.762). Thromboembolic lesions were identified with selective angiography in 627 branches (108 segmental and 519 subsegmental) for the cCT group and in 641 branches (87 segmental and 554 subsegmental) for the UHRCT group. Table 2 presents patients background data, indicating no significant differences in baseline parameters between the groups.

**Table 2:** Patient Characteristics and Hemodynamic Parameters before BPA

	cCT (n=21)	UHRCT (n=21)	p value
Age (years)	66±14	65±14	0.831
Sex (female) (n)	16	15	0.726
Height (cm)	157±11	158±8	0.481
Weight (kg)	55 (45–67)	58 (48−65)	0.96
No. of BPA Session (n)	5 (4−8)	5 (4−7)	0.451
Hemodynamic parameters			
mPAP (mmHg)	39±8.6	36.9±9.7	0.463
sPAP (mmHg)	59.5±17.3	65.8±18.2	0.262
dPAP (mmHg)	22 (19−30)	20 (17−27)	0.23
PVR (dynes・sec・cm−5)	564 (397−896)	607 (414−831)	0.85

BPA, balloon pulmonary angioplasty; cCT, conventional CT; UHRCT, ultra-high-resolution CT; mPAP, mean pulmonary arterial pressure; sPAP, systolic pulmonary arterial pressure; dPAP, diastolic pulmonary arterial pressure; PVR, pulmonary vascular resistance

### Sensitivity and specificity of lesion detection by CTPA

**Figure 3:**
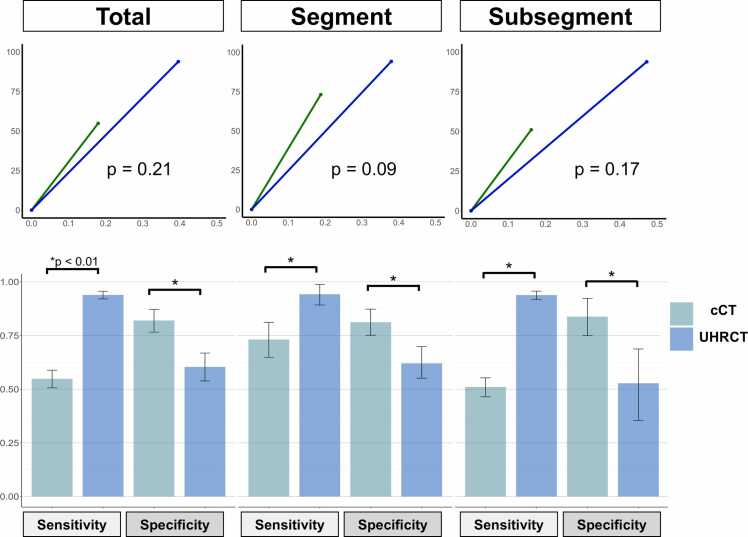
The sensitivity and specificity of CTPA, and FROC analysis. Overall, cCT demonstrated significant lower sensitivity and higher specificity compared to UHRCT (p < 0.01). Sensitivity in both segmental and subsegmental branches was also significantly higher in UHRCT, with a greater effect observed in subsegmental branches (p for interaction = 0.10). However, UHRCT had significantly higher false positive rate compared to cCT, resulting in no significant difference being observed in overall diagnostic performance when considering non-lesion detection (false positive) rates in FROC analysis. The FROC plot illustrates lesion detection sensitivity on the vertical axis and non-lesion detection (false-positive) rate on the horizontal axis.

Overall, cCT demonstrated a sensitivity of 54.6% (95% confidential interval (CI): 48.2 – 60.8) and specificity of 85.2% (95% CI: 75.6 – 91.4), whereas UHRCT demonstrated a sensitivity of 94.3% (95% CI: 91.9 – 96.1) and specificity of 60.2% (95% CI: 46.7 – 72.2) (Figure 3). The differences in sensitivity and specificity were 39.7% (95% CI: 31.0 – 47.8) and 25.0% (95% CI: 3.6 – 44.7), respectively, showing a significant difference (p < 0.01) (Figure 3).

**Figure 4:**
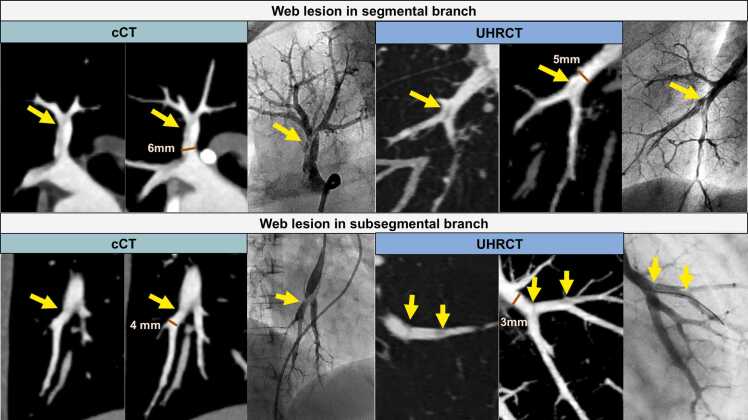
The representative cases of web lesions detected by each CT group in segmental and subsegmental branches. UHRCT provides a clearer depiction of web lesions, especially in subsegmental branches.

The sensitivity and specificity of cCT were 73.6% (95% CI: 63.3 – 81.8) and 85.0% (95% CI: 73.0 – 92.2) in segmental branches, and 50.5% (95% CI: 42.8 – 58.2) and 87.8% (95% CI: 71.7 – 95.3) in subsegmental branches, respectively. Those of UHRCT were 94.5% (95% CI: 86.8 – 97.8) and 61.7% (95% CI: 46.1 – 75.3) in segmental branches and 93.9% (520/554, 95% CI: 91.8 – 95.7) and 52.8% (19/36, 95% CI: 36.4 – 70.0) in subsegmental branches (Figure 3). UHRCT demonstrated a greater effect for sensitivity in subsegmental branches than segmental branches (p for interaction = 0.11). The FROC analysis showed that cCT demonstrated a higher lesion detection sensitivity than UHRCT (Figure 3). However, the difference in lesion detection sensitivity at 0.1 of non-lesion detection (false positive) rate was 7.3 and not statistically significant (95%CI: −2.9 – 20.2). Similarly, FROC analysis in segmental and subsegmental branches showed no significant differences, with differences of 14.9 (95% CI: −0.4 to 35.5) and 12.6 (95% CI: −3.6 to 33.7), respectively. Figure 4 shows representative cases with web lesions in segmental and subsegmental branches in each group.

### The agreement rate with selective angiography in lesion classification

**Figure 5:**
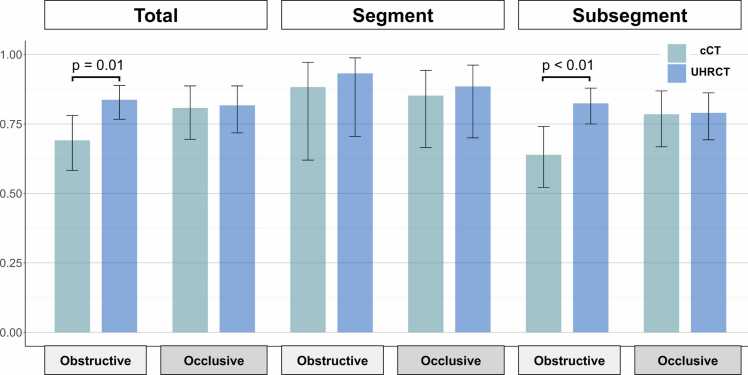
The agreement rate with selective angiography in lesion classification**.** UHRCT showed a higher agreement rate with selective angiography in classifying of obstructive lesions in total and subsegmental branches, indicating that UHRCT classified detected lesions as obstructive more accurately.

For the lesions detected by CTPA, cCT showed an agreement rate of 69.1% (95% CI: 58.3 – 78.1) in classifying obstructive lesions, while UHRCT showed a significantly higher agreement rate of 83.7% (95% CI: 76.7 – 88.9), indicating that UHRCT classified obstructive lesions more accurately (p = 0.01) (Figure 5). In segmental branches, the agreement rates were 88.3% (95% CI: 62.0 – 97.2) for cCT and 93.2% (95% CI: 70.5 – 98.8) for UHRCT, whereas, in subsegmental branches, UHRCT demonstrated a significantly higher agreement rate of 82.4% (95% CI: 75.0 – 87.9) than cCT (63.9%, 95% CI: 52.2 – 74.1) (Figure 5). However, UHRCT demonstrated no greater effect for agreement rate in subsegmental branches compared to segmental branches (p for interaction = 0.44). The agreement rates for classifying occlusive lesions were comparable between cCT (80.8%, 95% CI: 69.5–88.7) and UHRCT (81.7%, 95% CI: 71.8 – 88.7) (p = 0.89). This finding remained consistent across both segmental and subsegmental branches (Figure 5).

### Analysis of undetected lesions, interobserver reliability and image quality

**Figure 6:**
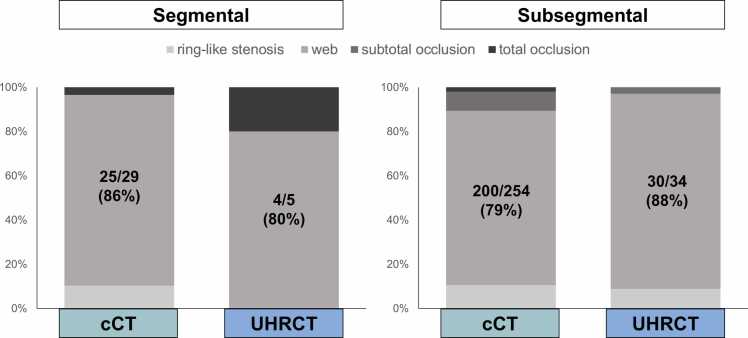
Morphological type of undetected lesions by CTPA. Of undetected lesions by CTPA, the web lesions accounted for 86% and 80% in the cCT and UHRCT in segmental branches, respectively. In subsegmental branches, web lesions accounted for 79% and 88% in cCT and UHRCT, respectively, indicating it is the most challenging lesion to detect with CTPA.

Web lesions accounted for 86% (25/29) in the cCT and 80% (4/5) in the UHRCT group in segmental branches. In subsegmental branches, web lesions accounted for 79% (200/254) in the cCT and 88% (30/34) in the UHRCT group, indicating web lesions are the most challenging to detect by CTPA (Figure 6). Based on the analysis of CTPA and selective angiography performed by the second observers, Cohen’s kappa for CTPA was 0.71 (95% CI: 0.67–0.76), with 718 agreements and 120 disagreements, indicating substantial interobserver agreement. For angiography, Cohen’s kappa was 0.51 (95% CI: 0.46–0.57), with 634 agreements and 203 disagreements, indicating moderate interobserver agreement. The intraobserver agreement for CTPA was κ = 0.67 (95% CI: 0.61–0.72), indicating substantial reproducibility in lesion detection over time.

The radiation dose of CTPA was significantly higher in the cCT group compared to the UHRCT group (CTDIvol: 8.2 [3.3–10.8] mGy vs. 5.5 [5.5–5.5] mGy, p<0.001); DLP: 300 ± 138 mGy-cm vs. 219 ± 23 mGy-cm, p=0.03). Although the main pulmonary artery attenuations showed no significant difference between the two groups (455 ± 143 Hounsfield units (HU) vs. 457 ± 127 HU, p=0.792), SD was significantly smaller in cCT group (21 [19−24] HU vs. 25 [24−30] HU, p<0.001). CT attenuation value in the ascending aorta was significantly lower in the cCT group compared to the UHRCT group (141 [84−193] HU vs. 311 [242−402] HU, p<0.001). The standard deviation of CT attenuation in air was significantly higher in the UHRCT group compared to the cCT group (9.8±1.2 HU vs. 11.4±2.4 HU, p ＝ 0.01).

## Discussion

The major findings of the present study were: 1) UHRCT significantly outperformed conventional CT (cCT) in lesion detection sensitivity, particularly in the subsegmental branches, while its specificity was lower, resulting in no significant difference in diagnostic performance between the two scanners when considering false positive; 2) UHRCT showed a higher agreement with selective angiography in classifying obstructive lesions; and 3) web lesions were the most difficult to detect on CTPA.

A recent meta-analysis demonstrated that high-quality CT studies had pooled sensitivity of 88% and specificity of 89% for CTEPH detection in segmental arteries-based analysis.^17^ High-quality CTPA images are crucial not only for diagnosing CTEPH but also for the assessment of PEA eligibility. However, BPA primarily targets subsegmental branches with diameters of 1 – 5 mm,^14^ requiring further enhancement of peripheral vasculature conspicuity in CTPA.^7^ This is the first study to focus on lesion detectability in subsegmental branches in CTPA compared to invasive selective angiography for BPA strategy. The results showed that cCT had low sensitivity for lesion detection especially in subsegmental branches, whereas UHRCT demonstrated higher sensitivity for both segmental and subsegmental branches. According to the European Respiratory Society, high-quality CTPA for CTEPH is defined by the following criteria: 1) pulmonary artery attenuation of 300–350 HU, 2) image reconstruction with a minimum slice thickness of 1 mm using a soft tissue kernel, and 3) high temporal resolution to ensure rapid thoracic coverage, minimizing breathing-related motion artifacts and venous contamination of the contrast medium.^9^ Considering the images from both the cCT and UHRCT met these criteria, spatial resolution was a critical factor influencing lesion detection in patients with CTEPH who predominantly have lesions in peripheral vessels. We have shown the effect of UHRCT in detecting lesions to be greater in subsegmental branches, which has been a known limitation of cCT, and thereby provide important insights into the indications for high spatial resolution CT. Since UHRCT and photon-counting detector CT became clinically available, extensive discussions have centered on how high spatial resolution CT can be integrated into clinical practice. Previous studies indicated that UHRCT facilitates non-invasive characterization of atherosclerosis by visualizing and quantifying calcified, stented or small diameter vessels, thus improving patient management and guiding treatment strategies.^12^, ^18^, ^19^, ^20^ In this study, UHRCT also showed a higher agreement rate in classifying obstructive lesions, especially in subsegmental branches. This lesion classification directly influences the success and complication rates of BPA,^7^ suggesting that UHRCT is promising for delineating and managing small but clinically significant anatomical structures and lesions, thereby improving clinical decision-making.

However, image noise is inversely related to spatial resolution and radiation exposure. The trade-off between sensitivity and specificity in UHRCT suggests that UHRCT may be more susceptible to false-positive findings possibly due to overinterpretation of subtle finding or increased image noise caused by the lower radiation dose and larger matrix size than cCT group. And FROC analysis suggests that despite the superior sensitivity of UHRCT, its ability to accurately detect lesions may not be significantly different from cCT when non-lesion detection (false positive) is considered. However, interobserver reproducibility was lower for angiography—despite being the gold standard for detecting peripheral lesions—compared to CTPA, even though both reviewers had substantial experience in evaluating angiographic findings in BPA. Therefore, the higher false positive rate observed in UHRCT may be due to its ability to detect lesions that angiography missed, such as mural thrombi and total occlusions. In terms of spatial resolution, the field of view is also crucial, suggesting that separate reconstruction of right and left lung images with a reduced display field of view may enhance diagnostic accuracy even with non-UHRCT scanners.^21^ In the cCT, it requires longer scan time and shorter injection durations to obtain the pulmonary perfusion iodine map avoiding the perfusion from systemic collaterals. These scan parameters in the cCT group could cause motion artifacts from heartbeats and respiratory motions, as well as impaired contrast opacification in peripheral vessels, reducing sensitivity for detecting peripheral lesions.^22^ We did not utilize the lung perfusion maps to identify lesions in cCT group, as our analysis focused solely on the morphological assessment of pulmonary arteries using CTPA. However, it may be valuable to investigate whether the combined use of lung perfusion imaging and morphological assessment could enhance lesion detection in future studies.

Web lesions, which result from thromboembolic material with subsequent scar formation,^23^ present as unique obstructive mesh-like filling defects in pulmonary arteries, differing from the arterial stenosis seen in arteriosclerosis. These web lesions are particularly challenging to detect by CTPA due to spatial resolution limitations and increased noise. However, the low specificity of UHRCT may be attributed to its ability to detect intramural thromboembolic materials and CTOs, which are not visible on angiography but detectable by CTPA (Supplemental Figure 2). This discrepancy may be attributed to subjective interpretation, contrast flow dynamics, or technical limitations in catheter-based imaging. However, comparing selective angiography findings before and after BPA, incorporating digital subtraction angiography or employing lesion assessment through consensus of two observers may improve interobserver reproducibility on angiography.

## Limitations

Our study had several limitations. First, it was a single-center observational study with a small sample size, thereby subjected to selection bias. Second, although all specialists independently reviewed the images in this study, given the low interobserver reproducibility, expert consensus reading can be considered as the reference standard. Third, not all branches were evaluated, because the series of BPA procedures were concluded based on individual improvements in hemodynamic parameters. Although, full right and left pulmonary angiography with contrast medium injection from the origins of each pulmonary artery can visualize all pulmonary branches simultaneously, detailed evaluation of subsegmental branches is limited due to overlap of branches, even when images are obtained from different angles. Fourth, blinding to scanner type during CT image assessment was not feasible due to the inherent differences in image characteristics between cCT and UHRCT, which may have introduced expectation bias. Finally, differences in scan and image reconstruction protocols existed between the two groups, although both met the guideline criteria for high-quality CTPA. The impact of matrix size on the lesion detectability in UHRCT requires further investigation. While larger studies are needed to access differences in clinical parameters, such as hemodynamics and complication, high-quality CT images provide detailed three-dimensional information on the vasculature, facilitating the selection of appropriate working projections and safe recanalization of occluded lesions during BPA.^10^ If future studies confirm the importance of high-resolution CT images for safe and effective BPA, usage of UHRCT could become more widespread.

## Conclusion

UHRCT demonstrated higher sensitivity in lesion detection than cCT, particularly for subsegmental branches, and improved lesion type classification in patients with CTEPH undergoing BPA. However, its lower specificity and increased false-positive rate highlight the need for cautious interpretation. Further research is needed to determine the clinical benefits of UHRCT in optimizing treatment strategies for BPA operators.

## Funding

This research was supported by the “Bayer Research Grant” from the Japan Radiological Society.

## Declaration of Competing Interest

Hidenobu Takagi, Hideki Ota and Kei Takase declare conflict of interest with Canon Medical Systems.

## Appendix A Supplemental material

Supplemental Figure 1 Distribution of evaluated branches in two groups. There is no significant difference in distribution of evaluated branches between the two groups (p = 0.762)

.

Supplemental Figure 2 A representative case of a chronic total occlusion identified by UHRCT but not clearly visualized on selective angiography**.** A chronic total occlusion in a subsegmental branch of the right A7 (yellow arrows) was difficult to detect on selective angiography prior to BPA. However, UHRCT clearly demonstrated the lesion, aided by anatomical reference to the adjacent bronchus. Based on the UHRCT findings, successful recanalization was achieved. MIP, maximum intensity projection
