# Supplementary figures and images for: Detectability of subsegmental lesions in patients with inoperable CTEPH: Comparison between ultra-high-resolution vs. conventional CT
Source: JHLT Open. 2025 Jul 18;10:100344. doi: 10.1016/j.jhlto.2025.100344 (PMC12362129; doi:10.1016/j.jhlto.2025.100344)

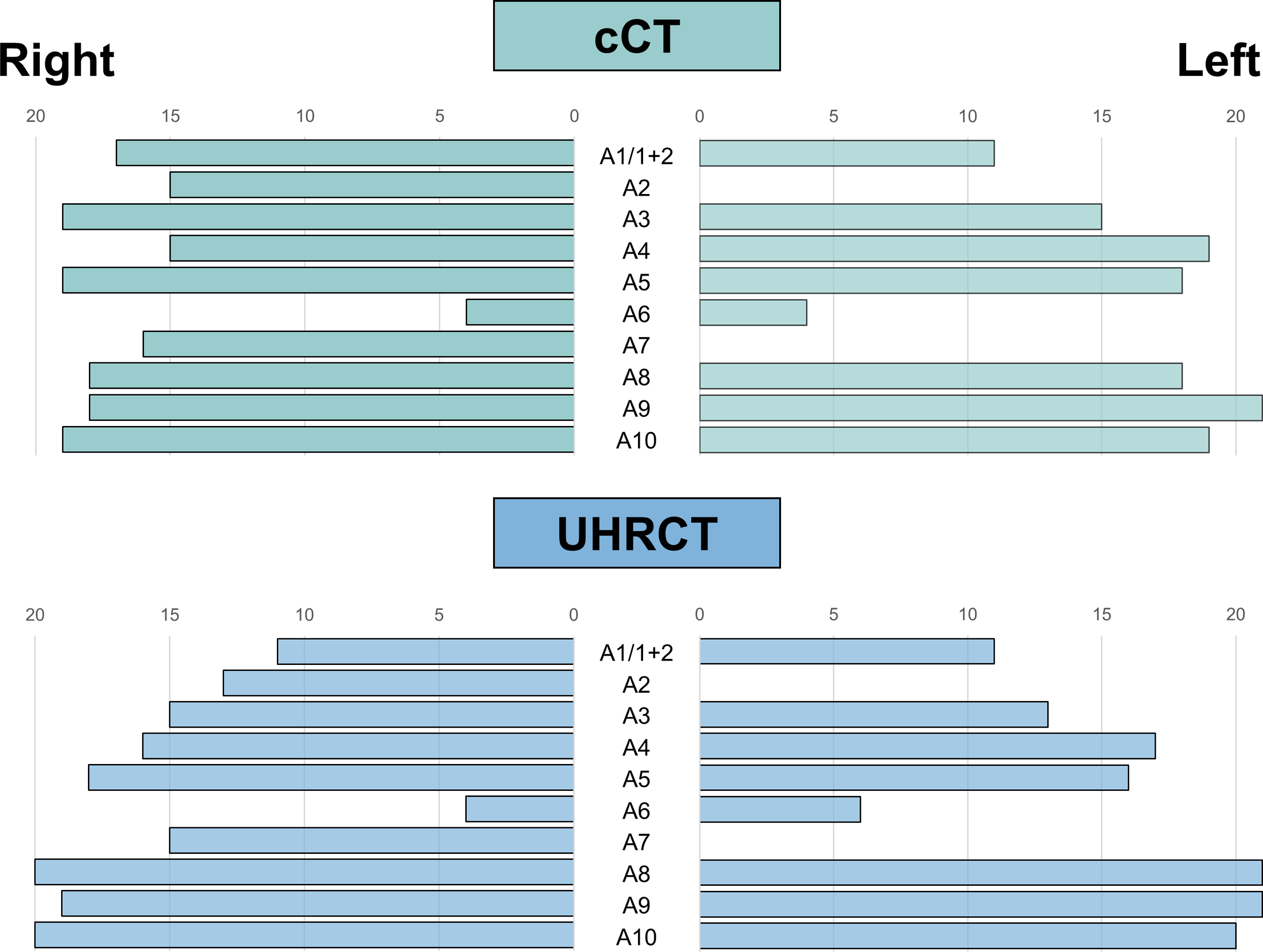

Supplement: Supplemental Figure 1 — Distribution of evaluated branches in two groups. There is no significant difference in distribution of evaluated branches between the two groups (p = 0.762) [file mmc1.jpg]

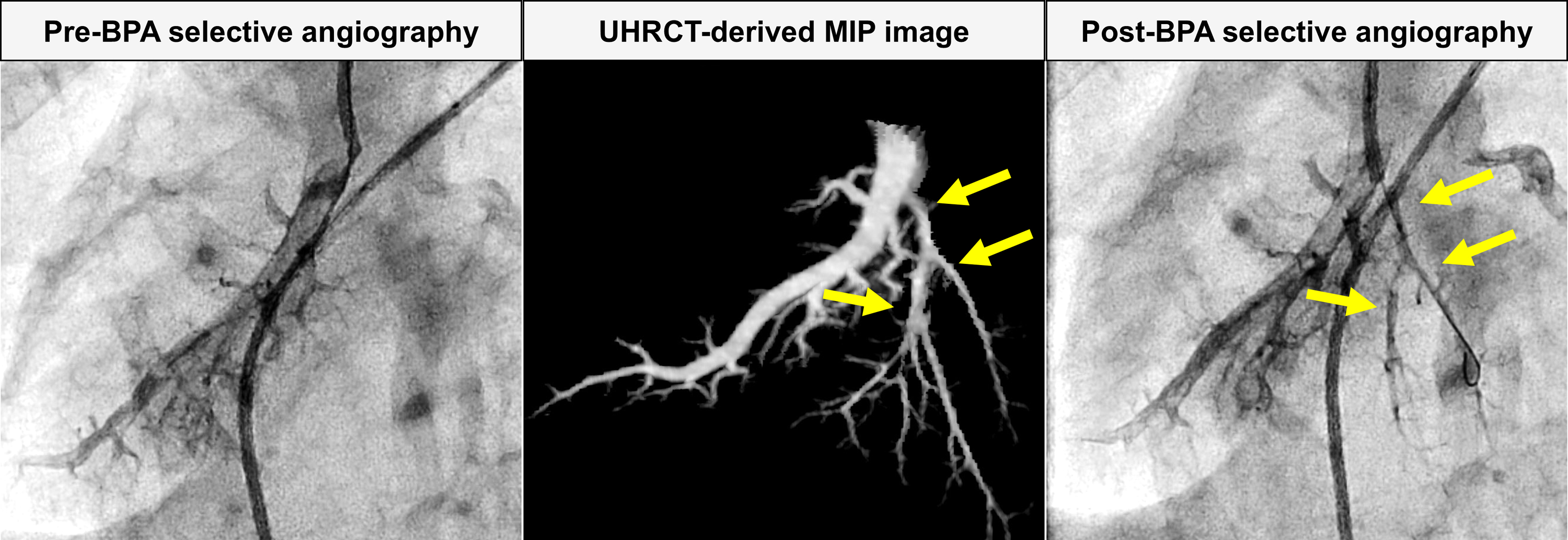

Supplement: Supplemental Figure 2 — A representative case of a chronic total occlusion identified by UHRCT but not clearly visualized on selective angiography. A chronic total occlusion in a subsegmental branch of the right A7 (yellow arrows) was difficult to detect on selective angiography prior to BPA. However, UHRCT clearly demonstrated the lesion, aided by anatomical reference to the adjacent bronchus. Based on the UHRCT findings, successful recanalization was achieved. MIP, maximum intensity projection [file mmc2.jpg]
